# Natural infection of Neotropical bats with hantavirus in Brazil

**DOI:** 10.1038/s41598-018-27442-w

**Published:** 2018-06-13

**Authors:** Gilberto Sabino-Santos Jr, Felipe Gonçalves Motta Maia, Ronaldo Bragança Martins, Talita Bianca Gagliardi, William Marciel de Souza, Renata Lara Muylaert, Luciano Kleber de Souza Luna, Danilo Machado Melo, Ricardo de Souza Cardoso, Natalia da Silva Barbosa, Marjorie Cornejo Pontelli, Priscila Rosse Mamani-Zapana, Thallyta Maria Vieira, Norma Maria Melo, Colleen B. Jonsson, Douglas Goodin, Jorge Salazar-Bravo, Luis Lamberti Pinto daSilva, Eurico Arruda, Luiz Tadeu Moraes Figueiredo

**Affiliations:** 1 Center for Virology Research, Ribeirão Preto Medical School, University of São Paulo, Ribeirão Preto, Brazil; 2 Department of Microbiology, Institute of Biomedical Sciences, University of São Paulo, São Paulo, Brazil; 3 Department of Ecology, São Paulo State University, Rio Claro, Brazil; 4 Department of Cell and Molecular Biology, Ribeirão Preto Medical School, University of São Paulo, Ribeirão Preto, Brazil; 5 Department of Biological Sciences, State University of Montes Claros, Montes Claros, Minas Gerais, Brazil; 6 Department of Parasitology, Institute of Biological Sciences, Federal University of Minas Gerais, Belo Horizonte, Brazil; 7 Department of Microbiology, National Institute for Mathematical and Biological Synthesis, Knoxville, Tennessee USA; 8 Department of Geography, Kansas State University, Manhattan, Kansas USA; 9 Department of Biological Sciences, Texas Tech University, Lubbock, Texas USA

## Abstract

Bats (Order: Chiroptera) harbor a high diversity of emerging pathogens presumably because their ability to fly and social behavior favor the maintenance, evolution, and dissemination of these pathogens. Until 2012, there was only one report of the presence of *Hantaviru*s in bats. Historically, it was thought that these viruses were harbored primarily by rodent and insectivore small mammals. Recently, new species of hantaviruses have been identified in bats from Africa and Asia continents expanding the potential reservoirs and range of these viruses. To assess the potential of Neotropical bats as hosts for hantaviruses and its transmission dynamics in nature, we tested 53 bats for active hantaviral infection from specimens collected in Southeastern Brazil. Part of the hantaviral S segment was amplified from the frugivorous *Carollia perspicillata* and the common vampire bat *Desmodus rotundus*. DNA sequencing showed high similarity with the genome of *Araraquara orthohantavirus* (ARQV), which belongs to one of the more lethal hantavirus clades (*Andes* *orthohantavirus)*. ARQV-like infection was detected in the blood, urine, and organs of *D*. *rotundus*. Therefore, we describe a systemic infection in Neotropical bats by a human pathogenic *Hantavirus*. We also propose here a schematic transmission dynamics of hantavirus in the study region. Our results give insights to new, under-appreciated questions that need to be addressed in future studies to clarify hantavirus transmission in nature and avoid hantavirus outbreaks.

## Introduction

Hantaviruses (family *Hantaviridae*) are membrane-enveloped viruses. Their genomes are small and consist of three segmented negative single-stranded RNAs designated large (L), medium (M), and small (S). These segments encode the virus polymerase, glycoproteins (Gn and Gc) and the nucleocapsid (N) protein, respectively^1,2^. Within natural hosts, hantaviruses do not cause apparent pathogenic effects^3^. However, transmission to humans can lead to severe diseases and death^4–6^; especially in South America, where hantavirus lethality rate reaches more than 40%^7^.

Hantaviruses are predominantly rodent-borne pathogens, although insectivore-bat-associated hantaviruses have been reported in the last decade^6,8^. Until 2012, only two human pathogenic hantaviruses have been reported in bats. The first report found viral antigens and isolated Hantaan orthohantavirus (HTNV) from two broadly distributed insectivorous species: *Eptesicus serotinus* and *Rhinolophus ferrumequinum*^9^. Recently, evidence of a lethal genotype of Andes orthohantavirus (ANDV), *Araraquara orthohantavirus* (ARQV), was documented by de Araujo *et al*.^10^ and Sabino-Santos Jr *et al*.^11^ among several Neotropical bats in Brazil. All the remaining reports of *Hantavirus* infection in bats involved genotypes not known to be pathogenic to humans.

Bats (order: Chiroptera) have highly social behavior that favors the maintenance, evolution, and dissemination of pathogenic viruses^12,13^. Bats are ranked second when considering top reservoirs of zoonotic viruses, and they host more zoonotic viruses per species than the first place order, rodents^14,15^. Therefore, the understanding of how these pathogens are transmitted and cause disease in humans is important in public health. Here, we show a systemic infection of hantavirus in Neotropical bats for the first time.

## Results

### Bats sampling and hantaviral detection

**Figure 1:**
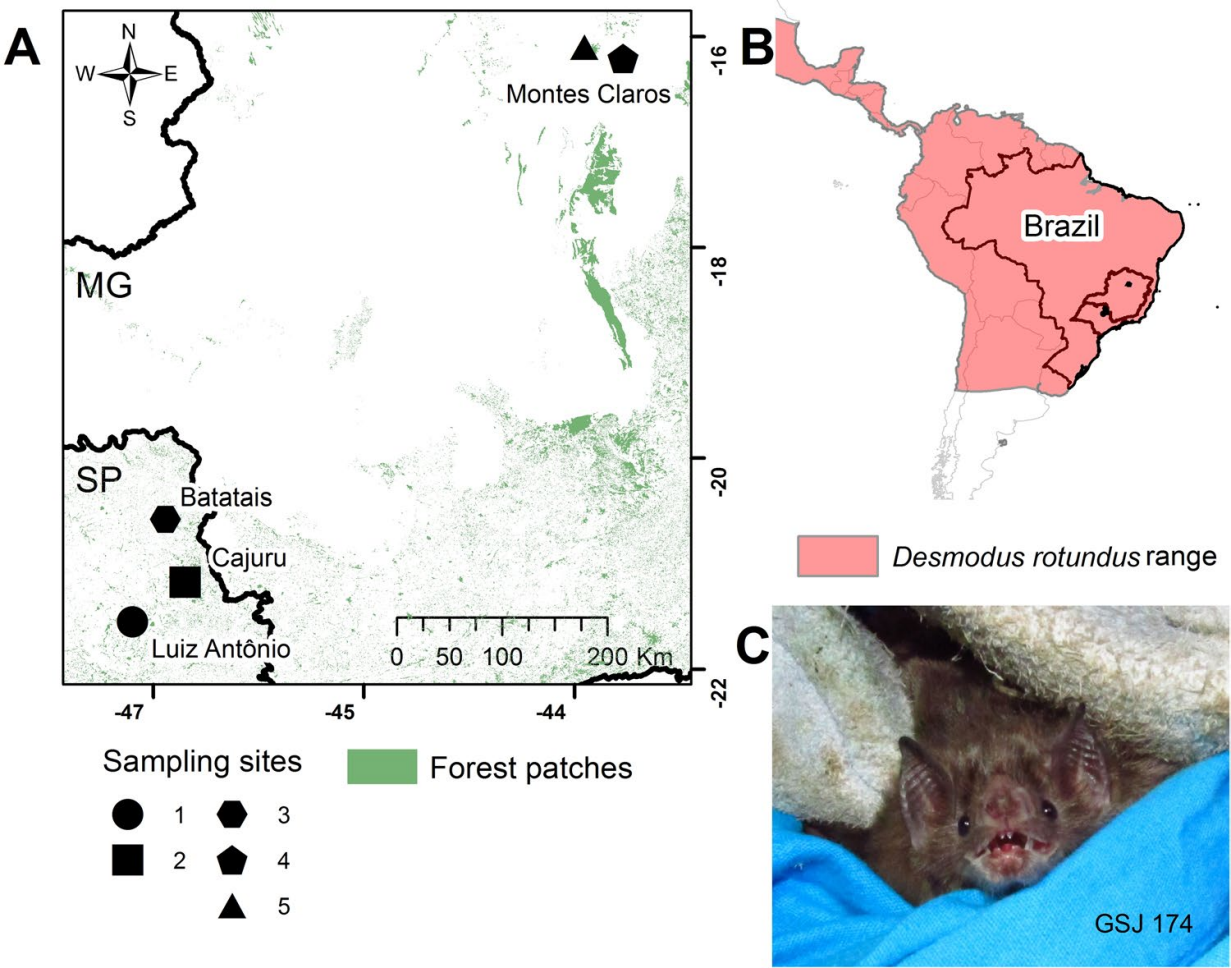
Study area, sampling points in Southeastern Brazil; *Desmodus rotundus* range and one tested bat. (**A**) The map highlights the five ecologically distinct trap sites in the Northeast of São Paulo State and North region of Minas Gerais State. In green, areas with native vegetation. (**B**) Distribution area of the common vampire bat *D*. *rotundus*^34^. (**C**) Photo of *D*. *rotundus* captured and tested in the study labeled as GSJ174. Bat picture was taken by Maia FGM. This map was created by Muylaert RL and Sabino-Santos Jr G, and generated using ArcGIS 9 version 9.1 (Environmental Systems Research Institute, USA).

We caught 270 bats from February 2012 to April 2014 with a sampling effort of 56,160 m^2^h, in southeastern Brazil (Fig. 1). In accordance with legal and ethical statements, whole blood from 53 of the 270 captured bats were screened for antibodies by indirect ELISA using a recombinant N protein of ARQV as antigen^11,16,17^. Nine phyllostomids bats representing seven species had antibodies to hantaviral N protein antigen and were selected for RT-PCR: *Artibeus lituratus* (n = 1), *A*. *obscurus* (n = 1), *A*. *planirostris* (n = 1), *Carollia perspicillata* (n = 1), *Chiroderma villosum* (n = 1), *Chrotopterus auritus* (n = 1), and *Desmodus rotundus* (n = 3) (for more details see Sabino-Santos Jr *et al*.)^11^. Blood, excreta (feces and urine), saliva and organs (heart, liver, kidneys, lungs, and spleen) of all seropositive animals had RNA extracted and screened. Hantaviral RNA was detected only in the blood of two phyllostomids species: the frugivorous *Carollia perspicillata* and the sanguivorous common vampire bat *Desmodus rotundus* (Figure S2 and Table S1). In the vampire bat, we amplified a partial S-fragment from the urine, heart, liver, lungs, spleen, and kidneys (Table S1 and Figure S1). Both bats were captured in a riparian forest surrounded by sugarcane monoculture in Batatais County, at sampling point number 3 (Fig. 1A).

### Evidence of active hantaviral infection

**Figure 2:**
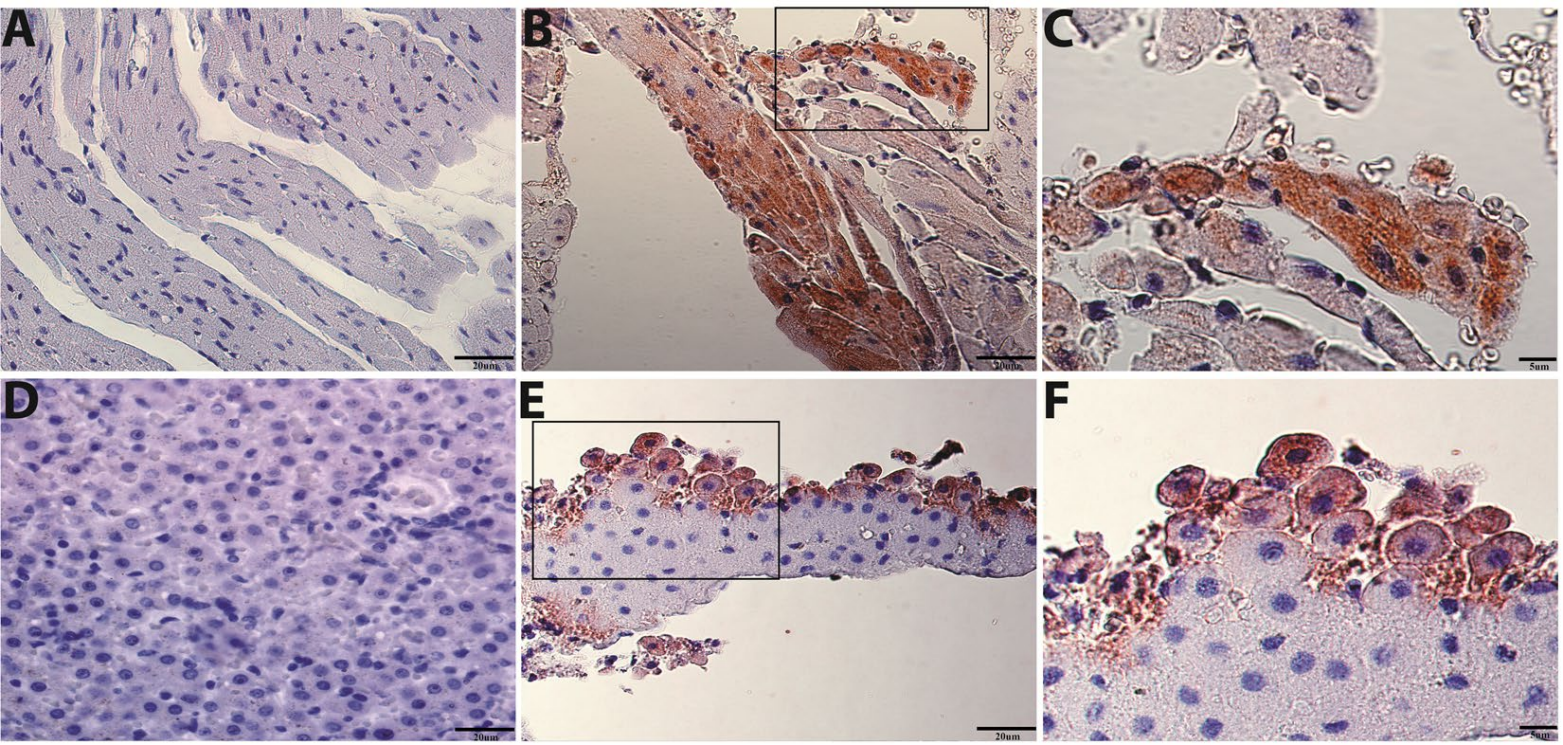
Immuno-histochemistry (IHC) hantavirus-infected tissues of *Desmodus rotundus*. (**A**) Fragment of heart tissue, used as negative control, from a non-infected *D*. *rotundus* immune-staining for the virus nucleocapsid in striated cardiac muscle cells. (**B**) Fragment of heart tissue of *D*. *rotundus* showing positive brown immune-staining for the virus nucleocapsid in striated cardiac muscle cells. (**C**) Represent inset of the same heart section in a higher magnification of a definite area from Fig. 2B. (**D**) Fragment of liver tissue, used as negative control, from a non-infected *D*. *rotundus* immune-staining for the virus nucleocapsid in hepatocyte cells. (**E**) Liver tissue of *D*. *rotundus* showing positive brown immune-staining for the hantavirus nucleocapsid in hepatocyte cells. (**F**) Represent inset of the same liver tissue section in a higher magnification of a positive area in Fig. 2E. IHC was counterstained with hematoxylin.

**Figure 3:**
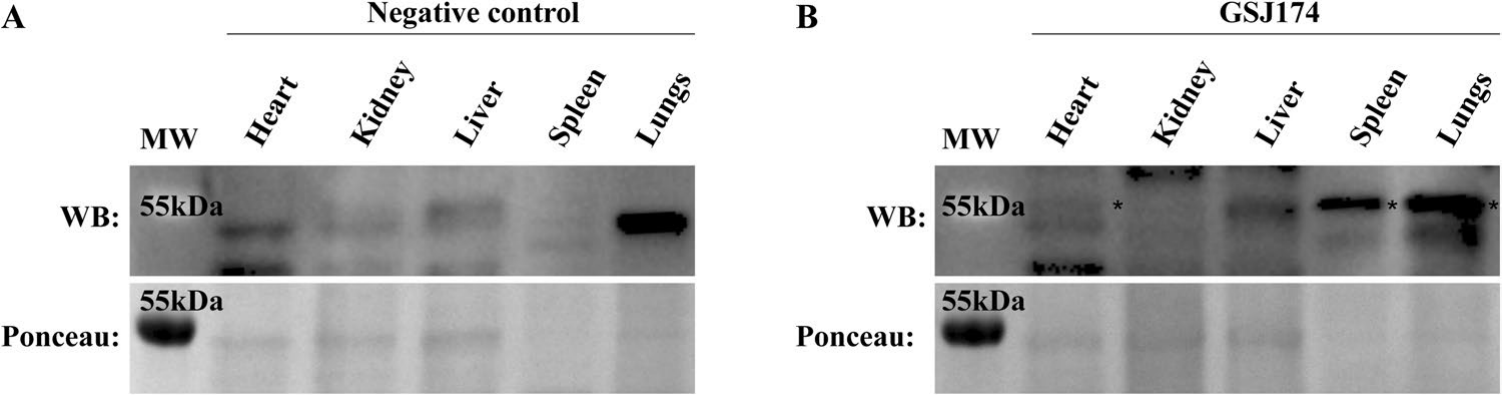
Organs tissues lysate from *Desmodus rotundus*.(**A**) Negative control and (**B**) infected organs were lysed as described in Material and Methods and submitted to western blot analyses. Tissue samples were equalized according to the total amount of protein shown by Ponceau (lower pannels) and viral protein was detected using a polyclonal anti-hantavirus hyperimmune murine ascitic fluid against N protein of ARQV (upper panels). *is showing detection of N protein in infected sample.

We assayed all organ tissues of the Neotropical bats *C*. *perspcillata* and *D*. *rotundus* by an immuno-histochemistry assay (IHC) detecting the viral nucleocapsid protein. Active infection was suggested in the organs of only *D*. *rotundus*. The monoclonal antibody to N protein of HTNV showed an intense cytoplasmic staining in myocardial cells and hepatocytes (Fig. 2B,C,E and F). Organ fragments of *Desmodus rotundus* were lysed and submitted to western blot (as described in Material and Methods section). Hantavirus nucleocapsid protein, the most synthesized viral protein, was found in all tested organs (except kidneys and liver) from the infected vampire bat, which indicates viral replication (Figs 3, S3).

### Phylogenetic relationship of hantaviruses detected

**Figure 4:**
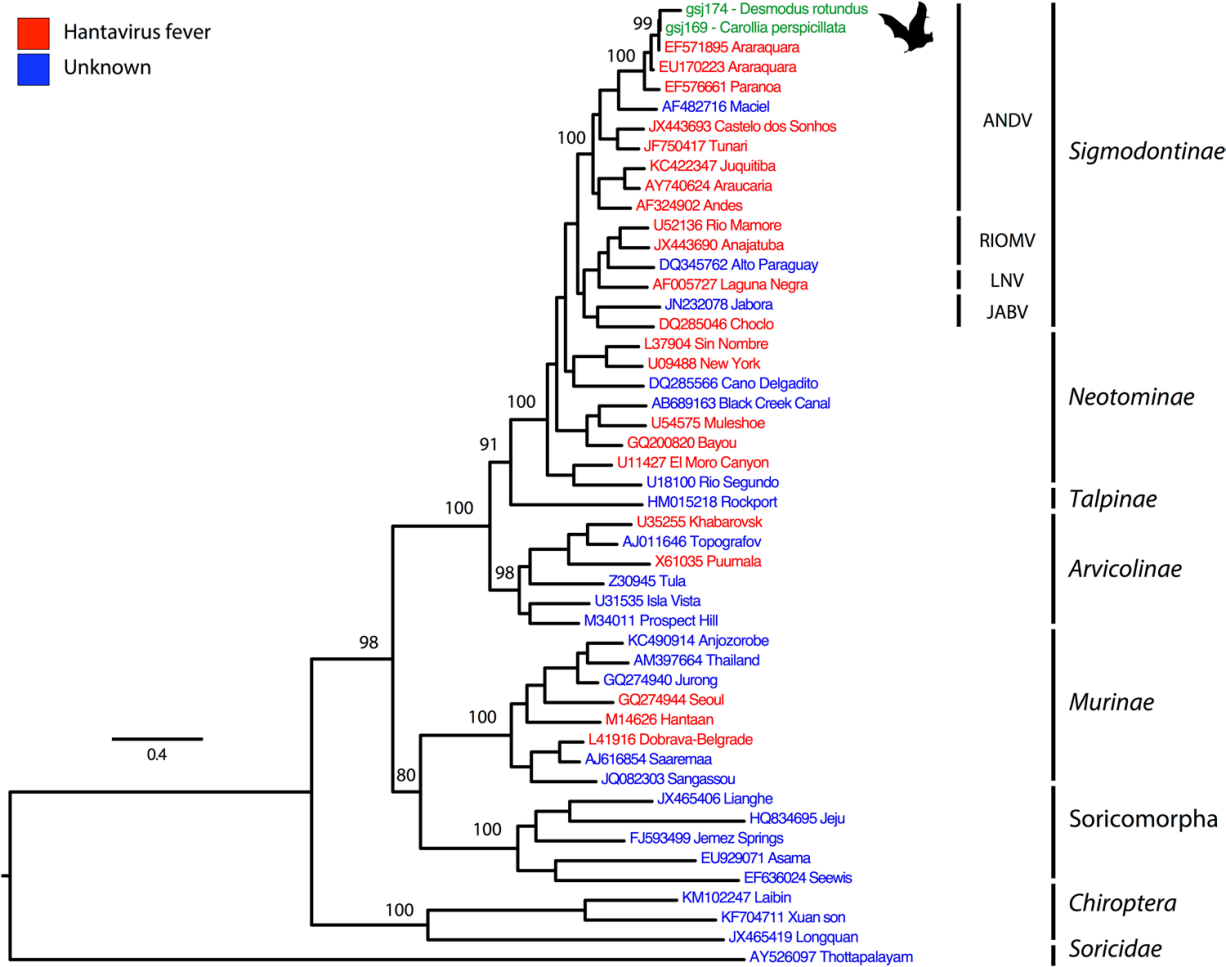
Maximum likelihood tree based on partial S segment nucleotides (~264 bp) showing evolutionary relationships of hantaviruses. The scale bar indicates evolutionary distance in numbers of substitutions per nucleotide substitutions per site, and the prime bootstrap support levels were designated. Phylogenies are midpoint rooted for clarity of presentation. Colors on hantaviruses branches indicate viruses described as human pathogen or unknown, as shown in the legend. Hantavirus sequenced in this study is highlighted with green color.

To determine phylogenetic relationships of the hantaviruses detected in *D*. *rotundus* and *C*. *perspicillata*, a phylogenetic tree based on partial S segment sequences was inferred using Maximum Likelihood (ML) (Fig. 4). In the tree, eight well-supported clades corresponded to already established hantavirus groups: Sigmodontinae, Neotominae, Arvicolinae, Murinae, Soricomorpha, Talpinae, Soricidae and Chiroptera^18^. Surprisingly, the phylogenetic analysis revealed that the strain detected in *C*. *perspicillata* and *D*. *rotundus* formed a monophyletic clade closely related to the ARQV variant of ANDV instead of grouping with the “Chiroptera clade,” as shown in Fig. 4. Additionally, the sequence from *D*. *rotundus* and *C*. *perspicillata* showed over 94% and 99% identity with ARQV respectively, compared to 43.4% averaged identity with hantavirus in the “Chiroptera clade” (Table 1).

**Table 1:** Distribution of identical nucleotide sites for the partial genome (~264 bp) of S segment.

Genbank	Virus	gsj169	gsj174^†^
EF57189	Araraquara orthohantavirus^*^	99.55%	93.72%
AF324902	Andes orthohantavirus	84.68%	79.23%
U52136	Rio Mamore orthohantavirus	82.43%	79.71%
L37904	Sin Nombre orthohantavirus	76.13%	73.71%
X61035	Puumala orthohantavirus	73.42%	68.60%
GQ274944	Seoul orthohantavirus	62.16%	59.42%
M14626	Hantaan orthohantavirus	63.96%	62.80%
KM102247	Laibin orthohantavirus	62.16%	59.42%
KF704711	Xuan Son orthohantavirus	59.91%	54.11%
JX465419	Longquan orthohantavirus	57.75%	55.22%
AY526097	Thottapalayam orthohantavirus	56.76%	53.62%
MF094269	gsj169	—	93.72%

^*^This virus is a genotype of Andes orthohantavirus.

^†^GenBank accession number MF094267.

## Discussion

Viruses are the most abundant biological entities on the planet Earth, and those originating in animal world cause most of emerging diseases of humans^14,15,19^. Viruses from bats can become zoonoses that are transmitted to domestic animals and humans presumably due to their ability to fly vast distances disseminating these viruses^15^. In our study, we found hantaviral RNA closely related to ARQV in urine of the common vampire bat *Desmodus rotundus*. This vampire bat could be transmitting ARQV-like through contaminated aerosol of its urine that could be inhaled thus infecting other animals^20^. However, this transmission mechanism should be confirmed by isolation of the hantavirus from urine to investigate if the pathogen shedding by the vampire bat is viable and infectious^21^.

Lungs are a primary target of infection in rodents and humans since the first detection of HTNV in lung tissue of the striped field mouse (*Apodemus agrarius*) and bats^9,22^; although, hantaviruses can also be found in other organs^12,23,24^. In the present study, we detected the N protein in almost all organs of *D*. *rotundus*, particularly in heart and liver, indicating virus replication (Figs 2 and 3). Similarly, other studies have shown liver as a target of infection^12,25,26^. ARQV-like RNA was present in the blood of the frugivorous bat *Carollia perspicillata*, and in almost all organs and secretions of the common vampire bat *Desmodus rotundus* (Figures S1 and S2). This suggests that the vampire bat could be chronically infected, as observed in rodents and soricomorphs^6,20^.

**Figure 5:**
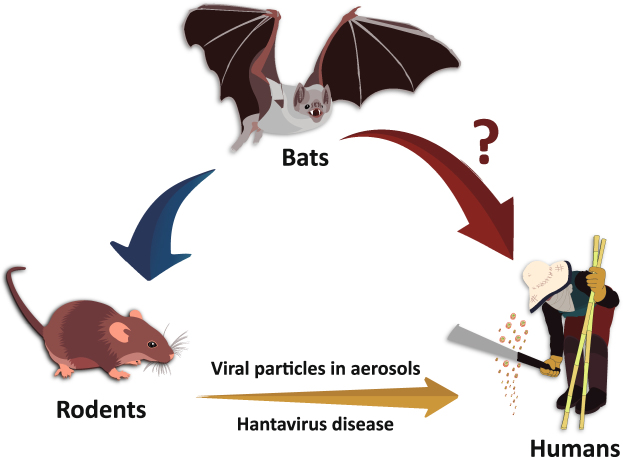
A schematic figure illustrating the transmission dynamics of hantavirus, in nature, in the study region, southeastern Brazil.

Although compelling evidence is provided that the vampire bat had viral RNA and antigen in many tissues, this could merely be a spillover infection from ARQV’s natural host, *Necromys lasiurus* (hairy-tailed bolo mouse). Guo *et al*.^18^ suggested that hantaviruses originated in bats and then dispersed among other mammalian reservoirs. However, due to the apparently common evolutionary history of bats with insectivores (shrews and moles), it is more likely that insectivores were the original reservoirs, and from these the virus has spread to bats and rodents^6,27,28^. There are reports of Sigmondontinae rodents infected in this same trap site region as the bats in the current study^7,29,30^. It is thought that small rodents may feed in the monocultures, but not necessarily nest in these monocultures. These rodents are more likely to live in the fragments where the vegetation is most preserved, thus sharing the same environment as bats, which depend on natural landscapes to survive environmental degradation^31–33^. *D*. *rotundus* feeds on blood of large and medium-sized mammals, including livestock, feral pigs, tapirs and red brocket deer. The bats potentially “take a ride” with the animals and disperse over long distances^34,35^. *D*. *rotundus* is broadly distributed over Latin America and in Brazil in particular, and considered commensal with humans and animals (Fig. 1B). Therefore, gathering our results with those already published, we propose a scheme illustrating the dynamics of transmission of hantavirus in nature in the study region (Fig. 5). In this way, as soricomorphs (shrews and moles) are not present in South America, infected bats could transmit hantavirus to rodents through their excreta. However, viremia of viruses in bats is believed to be low^36^. On the other hand, it is possible that hantaviruses are still in the stage of adaptation in rodents, as they still suffer a fitness cost^3^ in which case they would have become infected from bats as suggested by Guo *et al*.^18^. In our scenario, rodents would be acting as amplifying hosts where viruses would replicate, increase their load, and then be transmitted to humans via aerosols from their excreta (Fig. 5). Nonetheless, despite low viremia, direct transmission from vampire bats cannot be ruled out since they are in constant contact with humans (Fig. 5). Thus, there are still some questions that need to be addressed. More studies such as a large scale ecological and evolutionary survey of bats for ARQV may clarify how the virus managed to get into a vampire bat, how these bats transmit it, and ultimately if bats can serve as potential reservoirs of ARQV.

In short, we show herein that Neotropical bats *Carollia perspicillata* and *Desmodus rotundus* can be naturally infected with hantavirus in southeastern Brazil. In our survey, we found the circulating strain to be closely related to ARQV, one of the most virulent and lethal among all hantaviruses in humans. The N protein and/or the partial genome of an ARQV-like were detected in different organs and in the urine of the common vampire bat suggesting that this animal may play a role in hantavirus transmission and dissemination in the study region. These results show that bats are probably playing an under-appreciated part on the maintenance, circulation, and transmission of hantavirus in nature. This report expands the information available to public health authorities for establishing mitigation and surveillance strategies and understanding *Hantavirus* epidemiology and outbreak risks.

## Material and Methods

### Legal and Ethical Statements

Captured bats were handled following the guidelines of the American Society of Mammalogists for the use of wild animals in research and biosafety recommendations for working with animals potentially infected with hantavirus^37,38^. All procedures and protocols performed in the field as part of this study are in accordance with Brazilian environmental and wildlife protection laws and regulations. The study has been approved by the Brazilian Ministry of Environment (protocols 19838-5 and 41709-3). It has also been approved by the Ethics Committee for Animal Research of the University of São Paulo (020/2011) and the Federal University of Minas Gerais (333/2013). The research was approved by state laws (licenses IF-SP/COTEC 260108-007.043 and IEF-MG 012/2014). All laboratory procedures, until viral inactivation, were conducted in our BSL-3 + facility.

### Study areas and bat sampling

Bats were captured at five ecologically distinct sites in the northeast region of Sao Paulo State and northern region of Minas Gerais State, in southeastern Brazil (Fig. 1A). Site 1: Jatai Ecological Station, 21°37′ 16″S and 47°49′ 12″W, located at Luis Antonio county, encompasses an area of 9,000 ha and currently is the largest protected area in Sao Paulo State, with continuous *cerrado* vegetation (a Brazilian savanna-like vegetation) in addition to small enclaves of semideciduous forest patches. Site 2, located in Cajuru county, 21°16′ 31″S and 47°18′ 15″W, with most of the original vegetation (*cerrado*) converted into mono-specific cultivars. Site 3, located in Batatais county, 20°53′ 28″S and 47°35′ 06″W, is characterized by large sugar cane plantations with small patches of other types of vegetation. Site 4 was located at Sapucaia Ecological Park, which has an area of 37.66 ha and karst topography. Site 5 was located at Lapa Grande Ecological Station with an area of 9,600 ha, where a high concentration of caves and shelters can be found. Both sites 4 and 5 are located in Montes Claros county, 16°43′ 41″S and 43°51′ 54″W, and have a predominant vegetation of *cerrado* with patches of deciduous seasonal forest (dry forest), and stretches of transition to *caatinga* (a Brazilian desert-like vegetation). Field trips were conducted from February 2012 to April 2014. Places were sampled twice: in dry season (April to September) and in rainy season (October to March). We used 12 mist-nets in sites 1–3 and 6 mist-nets in sites 4 and 5 (model 716/12 P, 12 × 2,5 m; denier 75/2, 16 × 16 mm net; Ecotone, Gdynia, Poland). Bats were identified following standard ecological taxonomic keys^34,39,40^ and supplemented when needed by molecular data (e.g., based on a fragment of cyt-b gene)^41,42^. Vouchers specimens positive for hantaviral RNA, *D*. *rotundus* and *C*. *perspicillata*, were deposited in the Collection of Mammals from the Center for Taxonomic Collections from the Federal University of Minas Gerais, under numbers: 6948 and 6949, respectively. Saliva and excreta (feces and urine) were collected from all captured bats. According to ethical statements, only one specimen per species per trap-night could be anesthetized and euthanized. Thus, using Halothane ^**TM**^ (Sigma-Aldrich, USA) selected bats were anesthetized, and had blood collected by cardiac puncture. Next, bats were euthanized by deepening anesthesia and had organs collected: spleen, liver, kidneys, lungs, and heart. Organs were collected using sterilized tweezers, one for each specific organ to avoid cross-tissue contamination, washed with sterilized nuclease-free water. One-half of each sample was stored in cryovials with cell culture medium (DEMEM supplemented with 10% fetal calf serum, 1% antibiotic and antimycotic, and 15% nuclease free glycerol) for viral molecular screening, RT-PCR (Reverse Transcription-Polymerase Chain Reaction). The other half sample was placed into cryovials containing paraformaldehyde 4% (Merck, Germany), for immuno-histochemistry assays. All samples, except those in 4% paraformaldehyde, were flash-frozen in liquid nitrogen.

### RNA extraction, purification, and sequencing

The RNA was extracted from plasma, feces, urine, and tissues of bats with the QIAamp Viral RNA Mini Kit (QIAGEN, Germany), according to the manufacturer’s instructions. Tissues were homogenized in TissueLyser II (QIAGEN, Germany) as recommended by manufacturer. RNA extracts were quantified in NanoDrop ND1000 (USA) spectrophotometer. For cDNA synthesis, we used the High-Capacity cDNA Reverse Transcription Kit (Life Technologies, USA) following the manufacturer’s instructions. PCRs were performed according to Moreli and others^43^, for further purification and sequencing. PCR products were purified with ExoSap-TI (Affymetrix, USA) and used for nucleotide sequencing in an Applied Biosystems® - 3500 Genetic Analyzer (Thermo Fisher, USA).

### Control plasmids

Rio Mamore orthohantavirus (strain HTN-0007-TPV 4645) were amplified as previously described^43^. These PCR products (~ 324 bp and ~ 264 bp for Gn and S, respectively) were purified and cloned into pGEM-T Easy Vector (Promega Corporation, WI, USA), according to manufacturers’ guidelines, and used to transform DH10β *E*. *coli* cells. Colonies of bacteria were selected in LB medium supplemented with ampicillin 100 ng/ul and confirmed by PCR using same primers of product amplification. Plasmid preparation, from positive clone colonies, was isolated with Plasmid Mini Kits (QIAGEN, Germany) and used as positive controls for PCR reactions.

### Immuno-histochemistry assay

To determine the presence of the structural nucleocapsid protein of ARQV in tissue samples from PCR positive bats a protocol previously standardized was performed^44^. Tissues from bat organs fixed in 4% paraformaldehyde (Benchekroun *et al*., 2004) were dehydrated in increasing concentration ethanol solutions (50%, 70%, 80%, 90%, and 100%). Then, followed by xylene (1:1 xylene: ethanol and 100% xylene), embedded in paraffin, and 4 μm sections of tissue were obtained in a Leica microtome (Leica RM2125RTS, Heidelberger, BW, Germany). Slides were mounted with Entellan (Merck, Germany). Antigen retrieval in tissue slides was conducted by immuno-histochemistry. First, we used a monoclonal antibody (1:300 dilution in PBS/BSA 1%) against the N protein of HTNV (ab34757, Abcam plc, Cambridge, UK). The tissue in the slide had endogenous peroxidase blocked by 10% hydrogen peroxide and was incubated in humidified chamber at room temperature, for 60 min, washed with PBS, and incubated for 30 minutes with a biotinylated secondary horse antibody (1:300 dilution in PBS/BSA 1%) (BA-2000) (Vectastin® ABC Kit, Vector Laboratories, Burlingame, CA, USA) at room temperature (RT). The signal amplification was obtained by incubation with a polymer conjugated with streptavidin-peroxidase (s2438, Sigma-Aldrich, USA), at RT. Samples were counterstained with Harris hematoxylin (Vector). Positive staining was visualized by Nova Red peroxidase system with Vector Nova Red Substrate kit (SK-4800, Vector Laboratories, USA). Based on comparison with infected HeLa cells as positive control and uninfected heart tissues of *Desmodus rotundus* used as negative control, tissues showing red-brown cytoplasmic staining were considered as positive for hantaviral antigen.

### Western blot

Tissues were incubated in a lysis buffer [10 nM Tri-HCl (pH 8.0), 50 mM NaCl, 50 mM NaF, 1% Triton X-100, 0,1% SDS and 6 mM sodium deoxycholate] supplemented with a protease inhibitor mixture (Sigma-Aldrich, USA). Simultaneous disruption and homogenization were achieved through a high speed shaking of samples in micro-centrifuge tubes with stainless steel beads using TissueLyser II equipment (QIAGEN, Germany). Each of ten cycles of high-speed shaking, samples were followed by ice incubation for 1 min. The supernatant was collected after the lysates were centrifuged at 16.000 g for 20 min at 4 °C to remove tissue debris. Samples were mixed with loading buffer [4% SDS, 160 mM Tris-HCl (pH 6.8), 20% glycerol and 0,1% bromophenol blue] and boiled at 95 °C for 5 min. Proteins were resolved by SDS-PAGE and stained with Coomassie blue; and densitometry analyses of protein bands were calculated to be equalized to same amounts using Image Lab™ Software (Bio-Rad Laboratories, USA). Proteins were resolved by SDS-PAGE and transferred onto a nitrocellulose membrane (Millipore, USA), and blocked with PBS-T (PBS and 0,1% Tween 20) and non-fat dry milk, for one h. Primary antibody (1:500 dilution in PBS/BSA 1%), a hyperimmune murine anti-hantavirus ascitic fluid against the recombinant N protein of ARQV, was incubated overnight at 4 °C. After three washes with PBS-T, membranes were incubated with HRP-conjugated antibody anti-mouse (1:2000 dilution in PBS/BSA 1%) for one h at RT. After washing out the antibodies, proteins were detected by enhanced chemiluminescence ECL (GE Healthcare, USA).

### Phylogenetic analysis

Maximum likelihood (ML) phylogenetic tree was constructed using partial S sequences obtained from *Desmodus rotundus* (urine, blood, and heart) and *Carollia perspicillata* (blood) and an additional 47 nucleotides of S segment from representative hantaviruses. Multiple sequence alignment (MSA) was conducted by using combinations of Mafft v.7^45^ and Muscle v.3.7^46^. Phylogenies were inferred with IQ-TREE version 1.4.3 software that automatically obtained the best-fit model based on Bayesian Information Criterion. All segments were analyzed under the GTR + I + G4 model substitution model with 1,000 replicates^47^. Statistical supports for individual nodes were estimated using bootstrap value. The phylogenetic tree was visualized using the FigTree software v.1.4.2. The identity distance for the partial S segment was calculated using Genious 9.1.6 (Biomatters, Auckland, New Zealand). All sequences generated in this study can be retrieved from GenBank under accession numbers MF094267, for *D*. *rotundus* and MF094269 for *C*. *perspicillata*.

## Electronic supplementary material

Supplementary file
