# Supplementary material for: Natural infection of Neotropical bats with hantavirus in Brazil
Source: Sci Rep. 2018 Jun 13;8:9018. doi: 10.1038/s41598-018-27442-w (PMC5998146; doi:10.1038/s41598-018-27442-w)
Supplement: Supplementary file 1 — Supplementary file [file 41598_2018_27442_MOESM1_ESM.pdf]

Natural infection of Neotropical bats with hantavirus in Brazil

Gilberto Sabino-Santos Jr[1†], Felipe Gonçalves Motta Maia [1,2], Ronaldo Bragança Martins[1], Talita Bianca Gagliardi[1], William Marciel de Souza[1], Renata Lara Muylaert[3], Luciano Kleber de Souza Luna[1],Danilo Machado Melo[1], Ricardo de Souza Cardoso[1], Natalia Barbosa da Silva[4], Marjorie Cornejo Pontelli[1], Priscila Rosse Mamani Zapana[1], Thallyta Maria Vieira[5], Norma Maria Melo[6], Colleen B. Jonsson[7], Douglas Goodin[8], Jorge Salazar-Bravo[9], Luis Lamberti Pinto daSilva[4], Eurico Arruda[1] and Luiz Tadeu Moraes Figueiredo[1]

[1]Center for Virology Research, Ribeirão Preto Medical School, University of São Paulo, Ribeirão Preto, Brazil; [2]Department of Microbiology, Institute of Biomedical Sciences, University of Sao Paulo, Sao Paulo, Brazil; [3]Department of Ecology, Sao Paulo State University, Rio Claro, Brazil; [4]Department of Cell and Molecular Biology, Ribeirão Preto Medical School, University of São Paulo, Ribeirão Preto, Brazil; [5]Department of Biological Sciences, State University of Montes Claros, Montes Claros, Minas Gerais, Brazil; [6]Department of Parasitology, Institute of Biological Sciences, Federal University of Minas Gerais, Belo Horizonte, Brazil; [7]Department of Microbiology, National Institute for Mathematical and Biological Synthesis, Knoxville, Tennessee; [8]Department of Geography, Kansas State University, Manhattan, Kansas; [9]Department of Biological Sciences, Texas Tech University, Lubbock, Texas.

†corresponding author: Gilberto Sabino-Santos Jr (sabinogsj@usp.br/biosabinog@yahoo.com.br)

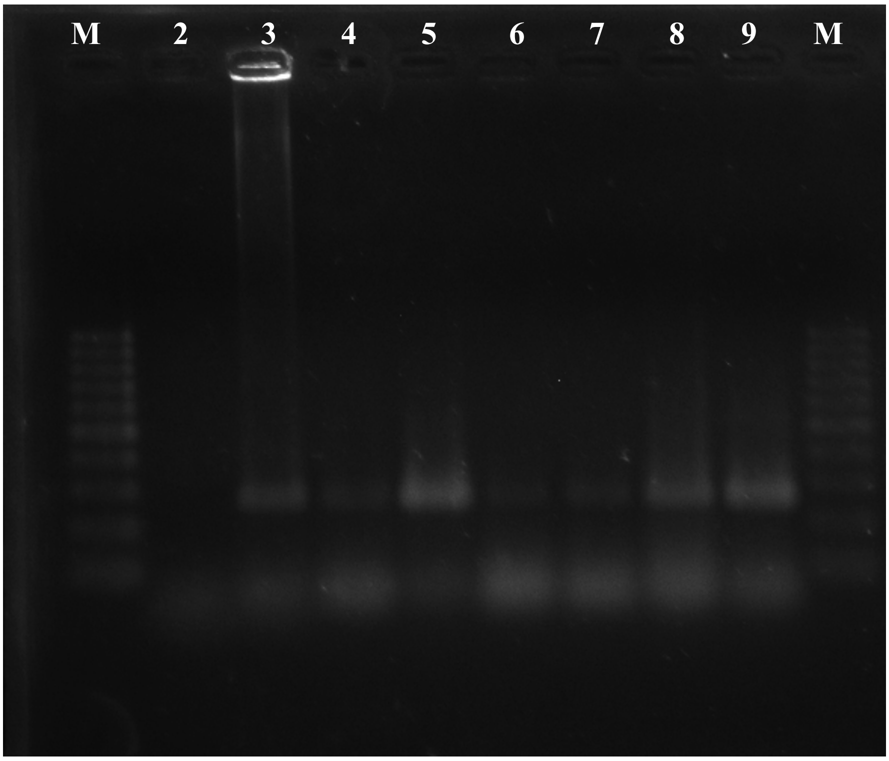

Figure S1.Result of PCR on GelRed-stained agarose gel 1.8% under UV light, displaying amplicons from the genomic S (gene N) segments of hantavirus from the vampire bat *Desmodus rotundus*, urine and tissues. Column M – molecular weigh as ladder of 100 bp; Colum 2 shows negative control for S segment; Columns 3, 4, 5, 6, 7 and 8 show ~264 bp of partial S segment from urine, spleen, heart, liver, lung, and kidney of *Desmodus rotundus* (sample GSJ 174), respectively; Column 9 shows partial S segment, ~264 bp, from Rio Mamore virus segments as positive control.

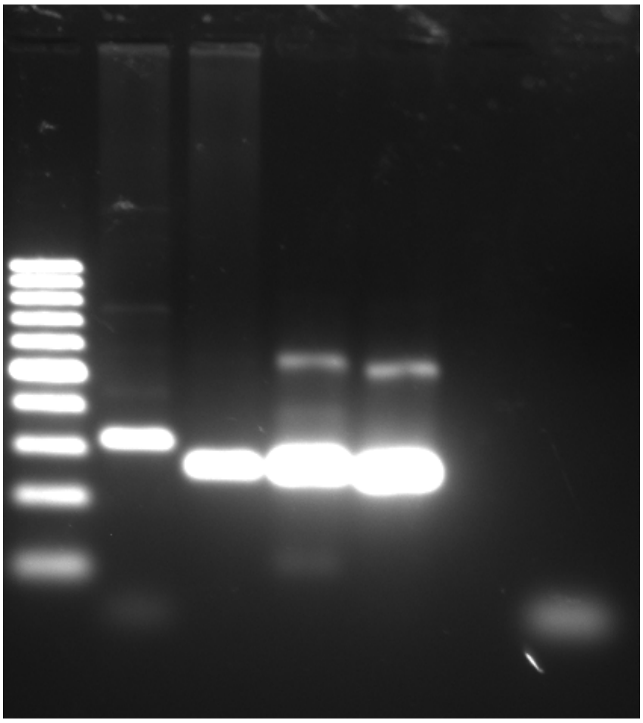

Figure S2. PCR result on GelRed-stained agarose gel 1.8% under UV light, displaying amplicons from the genomic M (Gn) and S (gene N) segments of hantavirus from blood of *Carollia perspicillata* and vampire bat *Desmodus rotundus*. Column M – molecular weigh as ladder of 100 bp; Columns 2 and 3 show partial M and S segment ~324 bp and ~264bp, respectively, from Rio Mamore virus segments as positive control. Columns 4 and 5 show partial S segment of ~264 bp from blood of *Carollia perspicillata* (GSJ 169) and *Desmodus rotundus* (GSJ 174). Column 6 is empty. Column 7 is showing negative control for hantavirus partial S segment of ~264bp.

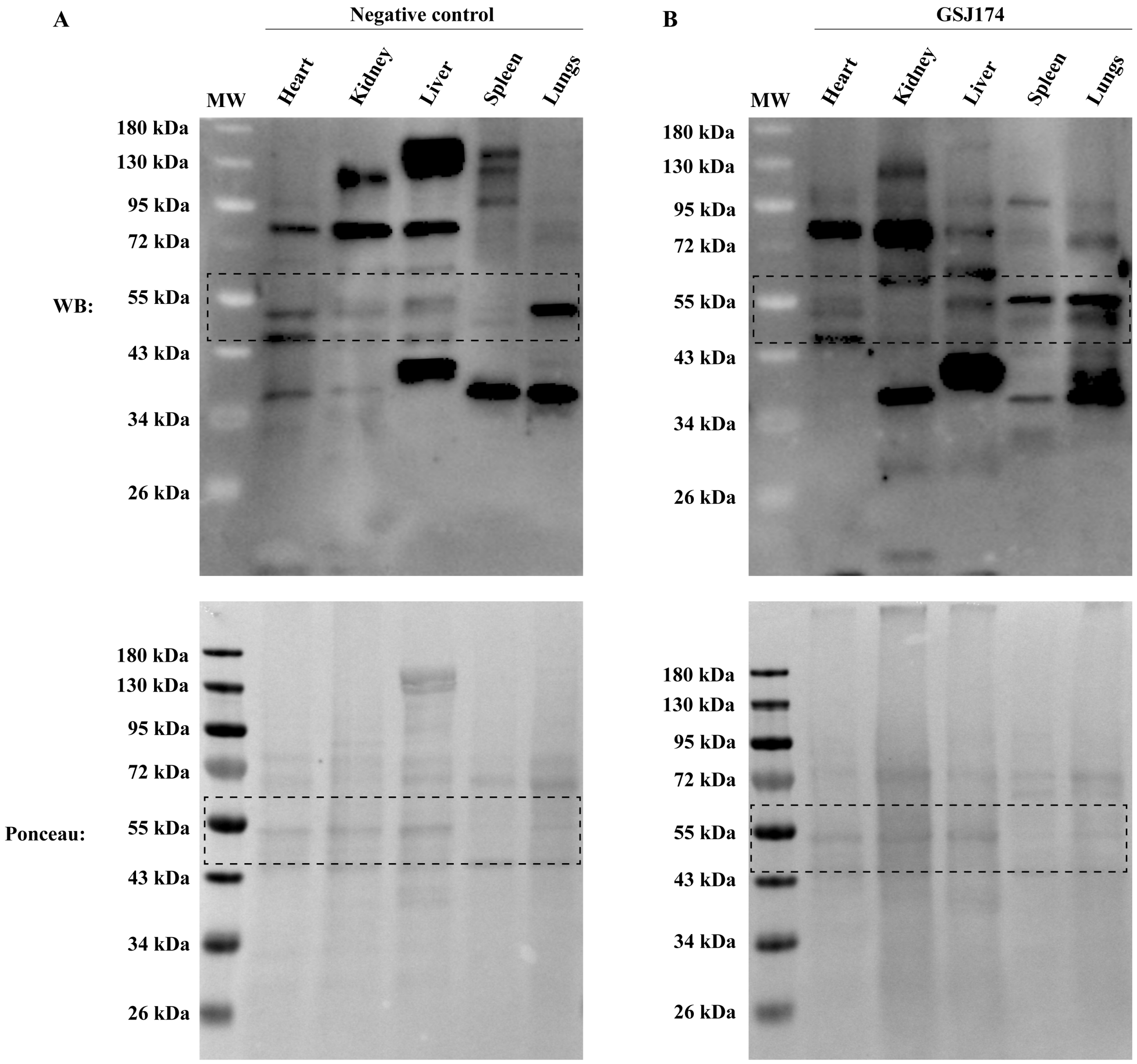

Figure S3. Organs tissues lysate from *Desmodus rotundus*. (A) Negative control and (B) infected organs were lysed as described in Material and Methods and submitted to western blot analyses. Tissue samples were equalized according to the total amount of protein shown by Ponceau (lower pannels) and viral protein was detected using a polyclonal anti-hantavirus hyperimmune murine ascitic fluid against N protein of ARQV (upper panels). Inset section is representing figure 3 in the main text.

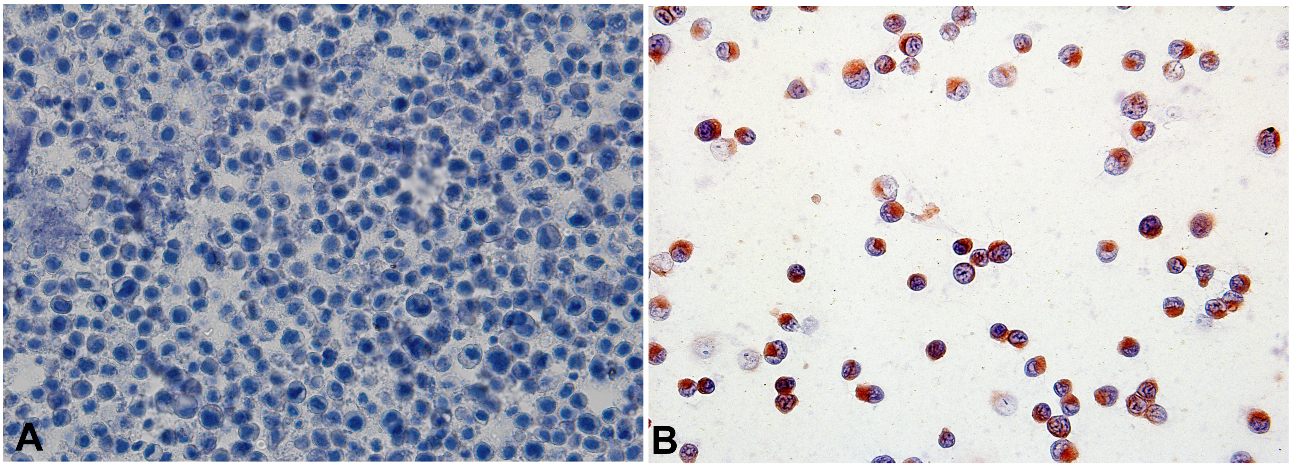

Figure S4. Immuno-histochemical staining of cells for the presence of hantaviral N protein. (A) Uninfected HeLa control cells. (B) Infected HeLa control cells with Rio Mamore virus (RIOMV, MOI = 1) used as positive control. In all IHC assay colour development for the virus nucleocapsid-binding antibody was performed with the Vector® NovaRed Peroxidase Substrate (Vector Laboratories, Burlingame, CA), which produces a red colour in the cytoplasm of infected cells. Counterstained with hematoxylin and magnificance of 400X.

Table S1. Positive samples for hantavirus infection among Neotropical bats.

Sample	Species positive for hantaviral infection (hantaviral Genome/tested)	
	Carollia perspicillata	*Desmodus rotundus*
Blood	1/10	1/5
Feces	0/10	0/5
Urine	0/10	1/5
Saliva	0/10	0/5
Heart	0/10	1/5
Lungs	0/10	1/5
Kidneys	0/10	1/5
Liver	0/10	1/5
Spleen	0/10	1/5
